# Hippopotamus (*H. amphibius*) diet change indicates herbaceous plant encroachment following megaherbivore population collapse

**DOI:** 10.1038/srep32807

**Published:** 2016-09-12

**Authors:** Kendra L. Chritz, Scott A. Blumenthal, Thure E. Cerling, Hans Klingel

**Affiliations:** 1 Global Change and Sustainability Center, University of Utah, Salt Lake City UT, USA; 2 Department of Anthropology, Graduate Center, City University of New York, New York NY, USA; 3 New York Consortium in Evolutionary Primatology, New York NY, USA; 4 Department of Geology and Geophysics, University of Utah, Salt Lake City UT, USA; 5 Zoologisches Institut, Universitaet Braunschweig, Braunschweig Germany.

## Abstract

Megaherbivores (>1000 kg) are critical for ecosystem health and function, but face population collapse and extinction globally. The future of these megaherbivore-impoverished ecosystems is difficult to predict, though many studies have demonstrated increasing representation of C_3_ woody plants. These studies rely on direct observational data, however, and tools for assessing decadal-scale changes in African ecology without observation are lacking. We use isotopic records of historical common hippopotamus (*Hippopotamus amphibius*) canines to quantify herbaceous vegetation change in Queen Elizabeth National Park, Uganda following a period of civil unrest and poaching. This poaching event led to population collapse of two threatened African megaherbivore species: hippopotamus and African elephants (*Loxodonta africana*). Serial carbon isotope ratios (δ^13^C) in canine enamel from individuals that lived between 1960–2000 indicated substantial increases in C_3_ herbaceous plants in their diet (<20% C_3_ in the 1960s to 30–45% C_3_ in the 80s and 90s), supported by other observational and ecological data. These data indicate megaherbivore loss results in succession of both woody and herbaceous C_3_ vegetation and further reaching effects, such as decreased grazing capacity and herbivore biodiversity in the area. Given multiple lines of evidence, these individuals appear to accurately capture herbaceous vegetation change in Mweya.

Megaherbivores exert a profound influence on ecosystem function and structure across ecosystems globally. They provide critical ecosystem services, such as nutrient cycling, soil transformation, increasing net primary productivity, maintenance of open environments and modification of trophic guild structure^1^,^2^. Yet, megaherbivores are threatened with extinction due to the effects of habitat loss, range contraction, competition with livestock, climate change, overharvesting and civil unrest^3^.

In African ecosystems, important threatened megaherbivores include the common hippopotamus (*Hippopotamus amphibius*) and African elephants (*Loxodonta africana*). Hippopotamus significantly engineer vegetation and environments near their aquatic habitats by feeding on ground-layer vegetation, transforming grassy areas into short-grass grazing lawns, and building water channels through areas near their habitat^3^. Hippopotamus feeding habits are unique in that they swing their heads back and forth close the ground, cropping vegetation closely with their lips; this method of feeding precludes them from consuming aquatic or woody vegetation in large numbers^4^. Likewise, elephants are important influencers of overall savanna habitat structure, function and diversity at multiple trophic levels^5^,^6^,^7^. Elephants browse selectively, and high elephant populations can lead to woodland decline and can result in the extirpation of woody plants in areas of intense elephant browsing pressure^5^.

These megaherbivores are critical to the health and maintenance of African savanna ecosystems. Savannas can be described as “mixed tree-grass systems characterized by a discontinuous tree canopy in a conspicuous grass layer”^8^ that can be defined structurally as having 5–80% fractional woody cover, which includes the structural categories grassland, wooded grassland and woodland/bushland/shrubland^9^,^10^,^11^,^12^,^13^. Encroachment of woody plants (“bush encroachment”; C_3_ shrubs and trees) in the grassy layer (C_4_ grasses) of savannas is an issue of major concern for ecologists, as increased shrub presence alters soil conditions and suppress grass productivity, affecting organisms at many trophic levels^14^. Studies on encroaching plants in savannas have focused predominately on C_3_ woody plants, and little attention has been given to the role of herbaceous non-grassy plants (C_3_ forbs and herbs). Replacement of grasses by herbaceous plants within savannas can be just as deleterious for ecosystem health and biodiversity as succession of woody vegetation, though the effects of herbaceous plant expansion within grassy biomes have understudied consequences^15^.

Evaluating the potential short and long-term effects on plant succession within ecosystems after megaherbivore collapse requires historical records of ecology from ecosystems where previous such population collapses have occurred. Comparisons are often drawn between the current rates of extinction and Pleistocene megafaunal extinctions, and though useful, those extinctions resulted in modern ecosystems for which there are no fossil analogs^3^. Building a historical record of ecology in African savannas to understand megaherbivore collapse and plant succession is difficult, as direct observations over decades have not been made for many savanna ecosystems. We provide a ground-truthed method for reconstructing terrestrial ecology on a decadal scale within African savanna ecosystems using stable carbon isotopes (*δ*^13^C) in hippopotamus canine enamel. This paper illuminates long-term ecological effects following population collapse of hippopotamus and elephants in a savanna ecosystem in Queen Elizabeth National Park (QENP), Uganda. A period of civil unrest during the regime of Idi Amin resulted in widespread poaching and near extirpation of elephants from the park during the 1970s. Historical diet records obtained from sequentially-sampled hippopotamus canine enamel for stable carbon isotopes indicate that herbaceous vegetation shifted such that these hippopotamus switched their diets from >80% C_4_ grass in the 1960s to as low as 55% C_4_ grass in the 80s and 90s on the Mweya Peninsula, previously one of the most diverse parts of the park. Data that indicates changes in the diets of these individuals accurately reflect both environmental changes and dietary shifts of the population at large, and are supported by other lines of evidence, including gut content studies, vegetation surveys and isotope studies of individuals from discreet time periods^16^,^17^,^18^,^19^. Though previous studies have used serially sampled hippopotamus enamel to reconstruct environments, none of these studies have had such data placed in the context of recorded environmental change^19^,^20^,^21^. Serial samples of *δ*^13^C from hippopotamus canines could serve as a useful measure of ecological change in other savanna parks where hippopotamus are present, and such skeletal collections exist.

## The ecological history of Queen Elizabeth National Park

**Figure 1:**
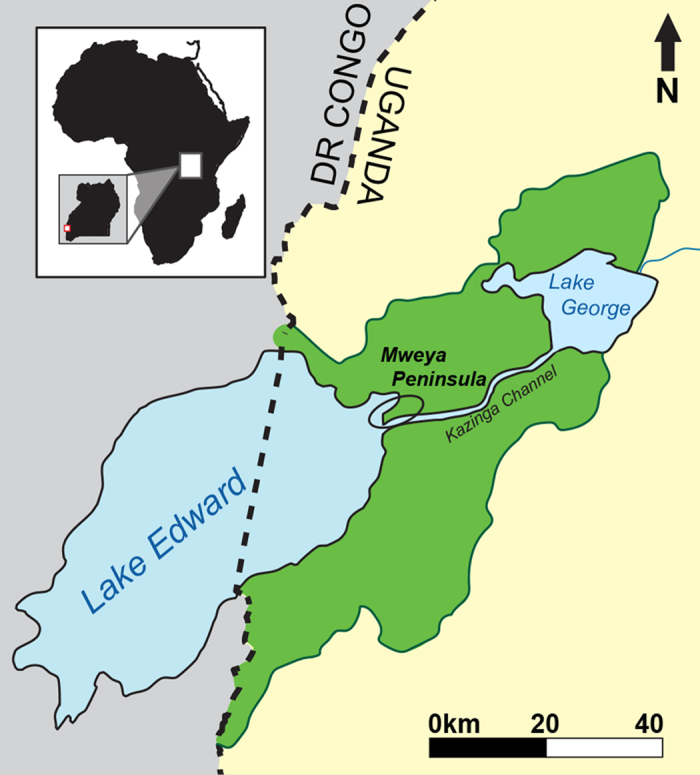
Map of Queen Elizabeth Park, Uganda. The red circle highlights the Mweya Peninsula. Image created in Illustrator (v. 19.2.0), re-drawn and modified from the image “Uganda_location_map.svg” downloaded from Wikimedia Commons (https://commons.wikimedia.org/wiki/File:Uganda_location_map.svg) and is licensed under the Attribution-ShareAlike 3.0 Germany license. The license terms can be found on the following link: https://creativecommons.org/licenses/by-sa/3.0/de/deed.en.

Queen Elizabeth National Park (Fig. 1) is located in western Uganda in the Albertine Rift Valley, along the border of the Democratic Republic of Congo (DRC). The park is 1,979 km^2^, surrounding Lakes Edward and George. Rainfall varies between 600 and 1400 mm/yr during March-May and in September-November^18^. The soils in the park are rich in volcanic ash, creating environments with high net primary production and many globally threatened and endemic plant and animal species^16^,^22^,^23^. At the time of census in the mid 1970s, QENP had the densest herbivore biomass (19,928 kg/km^−2^) on Earth^24^.

The effects of three major ecosystem drivers are elephant browsing, hippo grazing, and fire^23^. In the 1960s and 70s, hippos were observed to feed predominately in grasslands on or near Mweya (i.e., within a few kilometers of Lake Edward)^25^ that were maintained by elephant browsing and fire^5^,^6^,^26^. The dominant herbaceous vegetation on Mweya during that time was C_4_ grasses that grow in heavily grazed areas; trees and shrubs occurred in small thickets, clustered around emergent *Euphorbia candelabrum*^25^,^27^.

From 1972 to 1980, during the regime of Idi Amin, management of all national parks essentially ceased, and widespread poaching decimated herbivore populations across the country^26^,^28^. Intensification of poaching activities occurred in the late 1970s, resulting in collapsing herbivore populations and genetic bottlenecks^28^,^29^. The population of elephants within the park fell from 4,139 to ~150 individuals, and hippo populations fell to ~4,000 from nearly 12,000 individuals^28^.

Heavy wildlife poaching continued into the mid 1980s^28^, by which time there was a significant expansion in the size and number of woody vegetation, predominantly *Euphorbia candelabrum* and *Turraea robusta*^6^,^17^. Hippopotamus range restriction from poaching resulted in a positive-feedback for thicket encroachment: repeated trips onto land led to soil compaction, shunting rainwater into thickets and reducing fire fuel, further suppressing grass regrowth^25^,^30^,^31^.

### Stable Isotope ecology in African Mammals

Stable isotope analysis is a powerful tool for understanding aspects of mammalian herbivore ecology in Africa, and is particularly useful for generating ecological records on temporal and spatial scales that are difficult or impossible to observe^19^,^32^,^33^,^34^. Stable carbon isotope analysis (*δ*^13^C) of herbivore tooth enamel can reveal the relative proportions of C_3_ vs. C_4_ in the diets of African herbivores, and the relationship between *δ*^13^C_diet_ and *δ*^13^C_enamel_ values is well-understood in African ungulates^35^,^36^.

A common issue in modern ecological systems is generating continuous, decadal-scale historical records of environmental change. Serial samples of biological materials present a time-series of ecological information that can be useful as archives of environment, as each isotope sample represents an independent measure of diet and ecology for that individual^21^,^34^,^37^,^38^. Hippopotamus canines are excellent long-term ecological archives, since they are ever-growing and include isotopic input spanning ten or more years of enamel growth^36^,^39^,^40^. Therefore, stable isotope analysis of tusks is ideal for quantifying the nocturnal feeding behavior of hippos and thus ground-layer vegetation, and particularly for generating multi-year to decadal-scale ecological records from deceased animals. It is important to note, however, that these records only represent diet of individuals over a discreet time period, rather than of the population. Given the known behavioral characteristics of hippopotamus feeding behavior, we argue that these data can serve as a reflection of group feeding dynamics. Although hippopotamus travel alone to feed, their feeding areas are restricted and generally feed less than 6 km away from their aquatic habitats, often between 0.5–3 km, and tend to feed in the same areas as other hippopotamus^2^,^25^.

### Hippopotamus ecology and isotopic indicators of environment

The common hippopotamus is a large bodied herbivore found across Africa that lives in varied environments, ranging from arid savanna to forest^4^,^41^,^42^. They are semi-aquatic, spending the day resting in pools, rivers and lakes, and feeding nocturnally on terrestrial vegetation^43^. Hippos can significantly restructure grazing areas, mowing tall grasses into short, closely-cropped lawns. Early studies of hippo ecology suggested that hippos selectively feed on C_4_ grasses^16^,^44^. However, stable isotope ratios of carbon (^13^C/^12^C) in hippopotamus biological tissues have revealed considerable variability in the consumption of C_3_ plants (herbs and forbs) and C_4_ plants (tropical grasses < 3000 m), indicating a range in diets from purely C_3_-based to purely C_4_-based^19^,^45^,^46^. These data indicate that hippopotamus are more generalist feeders than previously thought, and dietary isotopic data reflect local herbaceous vegetation. Previous analysis of hippopotamus molars (integrating only a few years of growth) between 1970 and 1998 populations reveals statistically significant differences in diet between these two populations (ANOVA, Bonferroni’s test, *P* < 0.0001^47^). These molar analyses, however, only reveal snapshots of environmental change (i.e., an integrated measure of diet over the years of formation of the tooth) during two discreet time intervals. A more continuous measure of environmental change, such as those obtained by serial sampling of biological tissues, could better resolve when such changes took place.

## Materials and Methods

Lower hippopotamus canines were sampled from individuals who died on the Mweya Peninsula within Queen Elizabeth National Park. Date of death was assigned using recorded death dates (if known) or through radiocarbon dating of tusk enamel^39^. Calendar years for hippopotamus tusk samples were assigned using two methods. The 1960–1970 tusk was dated using bomb-curve radiocarbon dating^39^. The death years for the other two tusks were known–1991 and 2000. Using an average hippo lower canine growth rate of 4.4 cm/yr from five wild hippo tusks measured with bomb radiocarbon by Uno (2013), the calendar years were estimated for each sample in the profile assuming a constant growth rate.

Samples of enamel were drilled at intervals along the length of the tusk using a diamond-tipped drill bit and Dremel tool. Enamel powders were treated with 2% H_2_O_2_ for 30 minutes to remove organics, then washed 3 times with distilled water. Enamel samples were reacted with >100% phosphoric acid in a common acid bath in a dual-inlet Carboflo carbonate device. Stable isotope ratios (^13^C/^12^C and ^18^O/^16^O) of resulting CO_2_ were analyzed on an MAT 252 isotope ratio mass spectrometer, and stable isotope ratios are reported as delta (δ) values relative to the international carbon isotope standard, Vienna Pee Dee Belemnite (VPDB), following the standard permil (‰) notation, where *δ*^13^C = (R_sample_/R_standard_ − 1) × 1000. Enamel isotope values were corrected relative to an internal carbonate standard (Carrara Marble) calibrated to V-PBD and two in-house enamel standards (“MHS” and “MRS”). Dietary designations for hippos are given based on estimated dietary intake of C_4_ plants (lowland tropical grasses; −12.9‰^48^) and C_3_ plants (trees, shrubs, herbs, and forbs; −27.9‰^48^). This is calculated using the isotope enrichment factor (ε*):


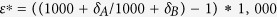


where ε^∗^ is the isotopic enrichment (*denoting compared materials not at equilibrium) between diet and herbivore tooth enamel (14.1‰)^35^, using the average isotopic value of modern C_3_ and C_4_ plants in eastern and central Africa^48^.

## Results

**Figure 2:**
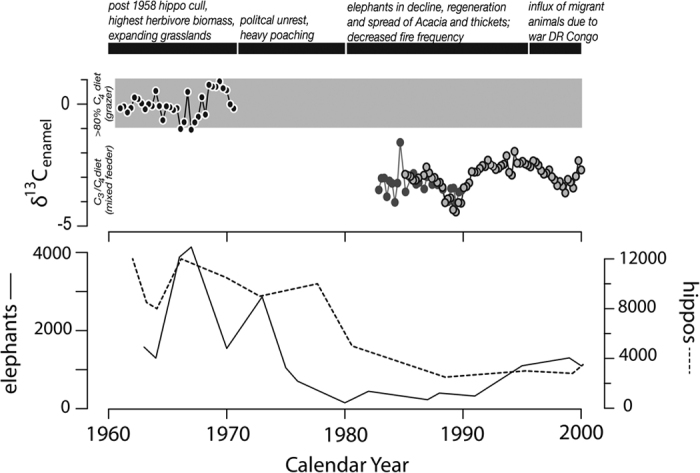
Carbon isotope profiles of the three Queen Elizabth hippo serial samples (1970 tusk in black, 1991 tusk in dark grey, 2000 tusk in light grey) and number of hippos (dotted line) and elephants (solid line) present in the park over time^23^^,^^56^. The chronolgy of major ecological and political events is listed above the graph.

The 1970s tusk reveals a diet of greater than 80% C_4_ grass intake from *ca*. 1960 to 1970 (Table 1, Fig. 2, SI Appendix Table 1). The 1991 hippo profile is significantly more depleted in *δ*^13^C than the 1970 profile, indicating a mixed C_3_/C_4_ diet (*ca*. 65% C_4_) from 1982 to 1991, following collapse of the elephant population in Queen (Fig. 2, SI Appendix Table 1). The third tusk includes the time interval from 1985 to 2000 with an estimated diet ranging from *ca*. 55 to 70% C_4_. Both the 1991 and 2000 individuals show local minima in *δ*^13^C values for the *ca*. 1988–1989 interval.

Hippopotamus canine carbon isotopes reveal a diet shift associated with increased consumption of herbaceous vegetation, which indicates a significant encroachment of C_3_ herbaceous plants within the Mweya Peninsula. Changes in atmospheric *δ*^13^C due to the burning of fossil fuels (the Suess Effect, <1‰) are smaller than changes in carbon isotopes from this dietary shift. In non-quantitative vegetation counts conducted in 1992 and 2009, Plumptre and others (2010) found that *Cynodon dactylon* (a C_4_ grass), *Commelina diffusa*, *Commelina africana* (C_3_ herbs), *Asystasia gangetica* (C_3_ forb), *Cyanotis foecunda* (a C_3_ flowering herb), *Achyranthes aspera* (C_3_ herb), *Ocimum suaveolens* (C_3_ herb), *Oplismenus hirtellus* (C_4_ grass) and two *Cyperus* species have become dominant, which suggests that hippopotamus have been increasingly relying on these C_3_ forbs and herbs. Though useful, it should be noted that these vegetation counts are not quantitative, and we present these data here as a validation for using hippopotamus diet change to infer ecological change.

## Discussion

Our findings reveal that hippopotamus have more flexible diets than previously thought, and can shift their diets to accommodate increased C_3_ herbaceous groundcover when preferred grasses are no longer present. Hippopotamus have traditionally been characterized as selective C_4_-grazers that feed on short grasses and sedges (which comprise 95–99% of their diet), supplemented by forbs^2^. This dietary classification was established by stomach content analysis by Field (1970), conducted on hippos in QENP during the 1960s, when grasses were abundant. However, modern hippopotamus environments are strikingly different from those of the 1960s, when groundcover vegetation on the Mweya Peninsula consisted predominately of grazing-tolerant C_4_ grasses^25^,^27^. Therefore, carbon isotopic records from serially sampled hippopotamus tusks may provide a much-needed quantitative resource for investigating historical ecological change across tropical grassy biomes in Africa.

The effects of poaching in the park have resulted in ecological restructuring of QENP. Aerial photographs and photomosaic analysis of vegetation types within the park between 1950 and 2006 indicate an increase in woody cover of ~30% across QENP^18^. Our findings demonstrate that in addition to bush encroachment, elephant poaching results in C_3_ encroachment in the herbaceous ground layer of savannas. The expansion of non-grassy ground vegetation restricts grazing capacity and decreases herbivore biodiversity and biomass, and these effects are intensified by the loss of hippopotamus-maintained grazing lawns^27^,^49^.

The long-term and wide-reaching effects of bush encroachment, such as changes in nutrient composition of soil, and %C and %N depletion in heavily encroached areas^50^, are further compounded by suppression of C_4_ grass growth, a critical resource for numerous mesoherbivores in savanna ecosystems. The combined effects of bush encroachment, which has already taken place across Africa^14^,^51^,^52^, and elephant poaching, which is occurring at unprecedented rates^53^, are likely to further exacerbate habitat degradation and suppress populations of grazing herbivores.

Civil war in eastern Democratic Republic of the Congo (DRC) in 1998 led to heavy poaching in Virunga National Park and other areas near QENP, resulting in herbivore migration to QENP and a partial recovery of herbivore communities, which may explain the delayed later succession of woody plants^54^. Elephant populations have been increasing in the park since 1990 (from ~500 to almost 3000 in 2005^23^), inhibiting only very recent (i.e., with the last 20 years) woody cover encroachment in the park, but not in the Mweya peninsula^23^. Although the deleterious effects of human conflict and wildlife poaching as a mechanism for ecosystem destabilization are known, further work is needed on the long-term ecological effects of overharvesting on ground vegetation in savanna ecosystems^55^.

## Conclusions

Megaherbivores are key ecological engineers that affect change at nearly every trophic level in African savannas, consuming and spreading nutrients on land and transforming soil properties and landscapes that increase environmental heterogeneity. Dietary stable isotope data from serially sampled hippopotamus canines from the Mweya Peninsula of QENP provide long-term ecological records that reflect increases in C_3_-vegetation, not only in woody layers as a result of elephant culling, but also in herbaceous ground layers. These isotopic data are supported by other lines of evidence for increasing C_3_ plant representation in the area. These data are critical for wildlife management within African savanna parks, as increasing C_3_ vegetation in both woody and herbaceous layers of savanna parks decreases grazing capacity for grass-feeding herbivores. The outcome of this ecological change is that grazers have been pushed to other parts of the national park, and even outside the park itself, posing serious threats for conservation in Africa. Our data suggest that hippopotamus canine isotopes could be used in savanna parks to build decadal records of ecological change in places where direct observational data are lacking.

## Additional Information

**How to cite this article**: Chritz, K. L. *et al.* Hippopotamus (*H. amphibius*) diet change indicates herbaceous plant encroachment following megaherbivore population collapse. *Sci. Rep.* **6**, 32807; doi: 10.1038/srep32807 (2016).

## Supplementary Material

Supplementary Information

## Figures and Tables

**Table 1:** *δ*^13^C values (vs V-PDB, mean and range) for the three hippo canines.

Specimen ID	Length (cm)	Date of death	Age determination	*δ*^13^C mean	*δ*^13^C range
KL	37	1970	radiocarbon	−0.03	−1.98
Queen VIC	35.5	1991	known	−3.34	−2.49
Q-09-KL	66.5	2000	known	−2.95	−2.48
